# Complete genome sequence of a clinical *Enterobacter asburiae* isolate harboring a novel variant of the carbapenemase gene, *bla*_IMI-24_

**DOI:** 10.1128/mra.00196-25

**Published:** 2025-06-18

**Authors:** Jun Kawase, Yuta Kawakami, Naoki Fujisawa, Hiroki Hayashi, Ryoji Nomura, Masahiro Kurahashi, Mieko Wada, Satowa Suzuki

**Affiliations:** 1 Division of Bacteriology, Shimane Prefectural Institute of Public Health and Environmental Science, Matsue, Shimane, Japan; 2 Division of Atmospheric Environment, Shimane Prefectural Institute of Public Health and Environmental Science, Matsue, Shimane, Japan; 3 Antimicrobial Resistance Research Center, National Institute of Infectious Diseases, Higashimurayama, Tokyo, Japan; California State University San Marcos, San Marcos, California, USA

**Keywords:** class A β-lactamases, IMI-type carbapenemases, Enterobacteriaceae, novel variant

## Abstract

*Enterobacter asburiae* strain CRE21025 was isolated from a gallbladder abscess of a patient admitted to the hospital in Shimane Prefecture in 2021. Here, we present the complete genome sequence of this isolate, bearing a novel variant of the imipenemase-type gene, *bla*_IMI-24_, by whole-genome sequencing using Oxford Nanopore and Illumina methods.

## ANNOUNCEMENT

Imipenemases (IMI), which inactivate carbapenems, belong to Ambler class A carbapenemases, with variants ranging from *bla*_IMI-1_ to *bla*_IMI-23_ reported as of December 2024 (1, 2). Here, we report the complete genome sequence of carbapenem-resistant *Enterobacter asburiae* strain CRE21025, harboring a novel IMI-type variant.

In 2021, *E. asburiae* strain CRE21025 was isolated from a gallbladder abscess sample from a hospitalized patient in a hospital laboratory in Shimane Prefecture, Japan, by culturing the sample on 5% sheep blood agar at 37°C for 48 h. The pure isolate was subsequently transferred from the laboratory to the first author’s institute, streaked onto Mueller-Hinton agar II (MHII) (Becton Dickinson, USA), and incubated overnight at 35°C. A single colony from MHII was inoculated into LB broth (Sigma-Aldrich, USA) supplemented with meropenem (8  µg/mL) (Sigma-Aldrich) and onto MHII and incubated overnight at 35°C. MHII cultures were subjected to the Carba NP test (3) and Etest (bioMérieux Japan). CRE21025 was confirmed to produce carbapenemase and was determined to be imipenem-resistant and meropenem-intermediate according to CLSI M100 2018.

Genomic DNA was extracted from the LB overnight cultures at 35°C using Genomic tip 100/G (Qiagen, Germany). Genomic libraries were prepared using the Illumina DNA Prep Kit (Illumina, USA) with 300–350 bp insert size and the Ligation Sequencing Kit SQK-LSK109 (Oxford Nanopore Technologies, UK) without size selection or shearing. Illumina and Nanopore sequencing was performed using the Illumina iSeq100 system with a 2 × 150 bp paired-end protocol and the Oxford Nanopore MinION MK1C instrument with flow cell R9.4.1 (FLO-MIN106D), respectively. Default parameters were used for all software except where otherwise noted. The Illumina raw reads were adapter trimmed with Local Run Manager software v2.4.0 (Illumina). Base calling and adapter trimming for Nanopore raw reads were performed using Guppy v5.1.12/MinKNOW v21.11.6 (Oxford Nanopore Technologies). Quality filtering, using Nanopore and Illumina reads after adapter trimming, was performed (quality score limit, 5%) with the Trim Reads tool in CLC Genomics Workbench v21.0.5 (CLC21.0.5) (Qiagen). Filtered Nanopore reads were assembled using CLC21.0.5 (Long-Read Support tool). The assembly graph from this tool indicated two circular contigs, which were polished using filtered Illumina reads with the same tool. The average coverage of two contigs was calculated by the Long-Read Support tool and Map Reads to Contigs tool. DFAST software (https://dfast.ddbj.nig.ac.jp) was used for gene annotation and the genome rotation to start with *dnaA*. The two contigs were analyzed using PlasmidFinder v2.1 (4), ResFinder v4.1 (5), and the online version of BLASTN (against the NCBI nucleotide database; BLAST DB v5) (https://blast.ncbi.nlm.nih.gov/Blast.cgi). Moreover, the average nucleotide identity (ANI) was calculated using Skani (6) within DFAST (7) against a reference data set of 17,715 species genomes (ver. 2025-02-05) obtained from NCBI data sets. The reads and sequence data are listed in Table 1.

**TABLE 1:** Sequencing data and genome characteristics of *Enterobacter asburiae* CRE21025

Category	Feature	Data for	
		Chromosome (EA21025)	Plasmid (pEA21025)
Assembly	No. of contig	1	1
	Structure of contig	Circular	Circular
	Total length of the genome (bp)	4,548,216	132,644
	GC content (%)	55.9	50.6
	Average coverage, Illumina (×)	74	163
	Average coverage, Nanopore (×)	110	518
	Predicted no. of coding DNA sequences	4,264	145
	rRNAs (5S, 16S, 23S)	25 (9, 8, 8)	0
	tRNAs	84	0
	Highest ANI against type strains	98.75% Against *Enterobacter asburiae* FDAARGOS_892 (accession: GCA_016027695.1)	No test
	Replicon type of plasmid	No test	IncFII
	Antibiotic resistance genes	bla_ACT-4_, fosA, oqxA, oqxB	bla_IMI-24_
	GenBank accession no.	AP029016	AP029017
Illumina data	No. of reads after adapter trimming	2,525,000	
	No. of reads after quality filtering	2,521,219	
Nanopore data	No. of reads (N_50_)	220,000 (18,373 bp)	
	No. of filtered reads (N_50_)	198,266 (12,431 bp)	

**Fig 1:**
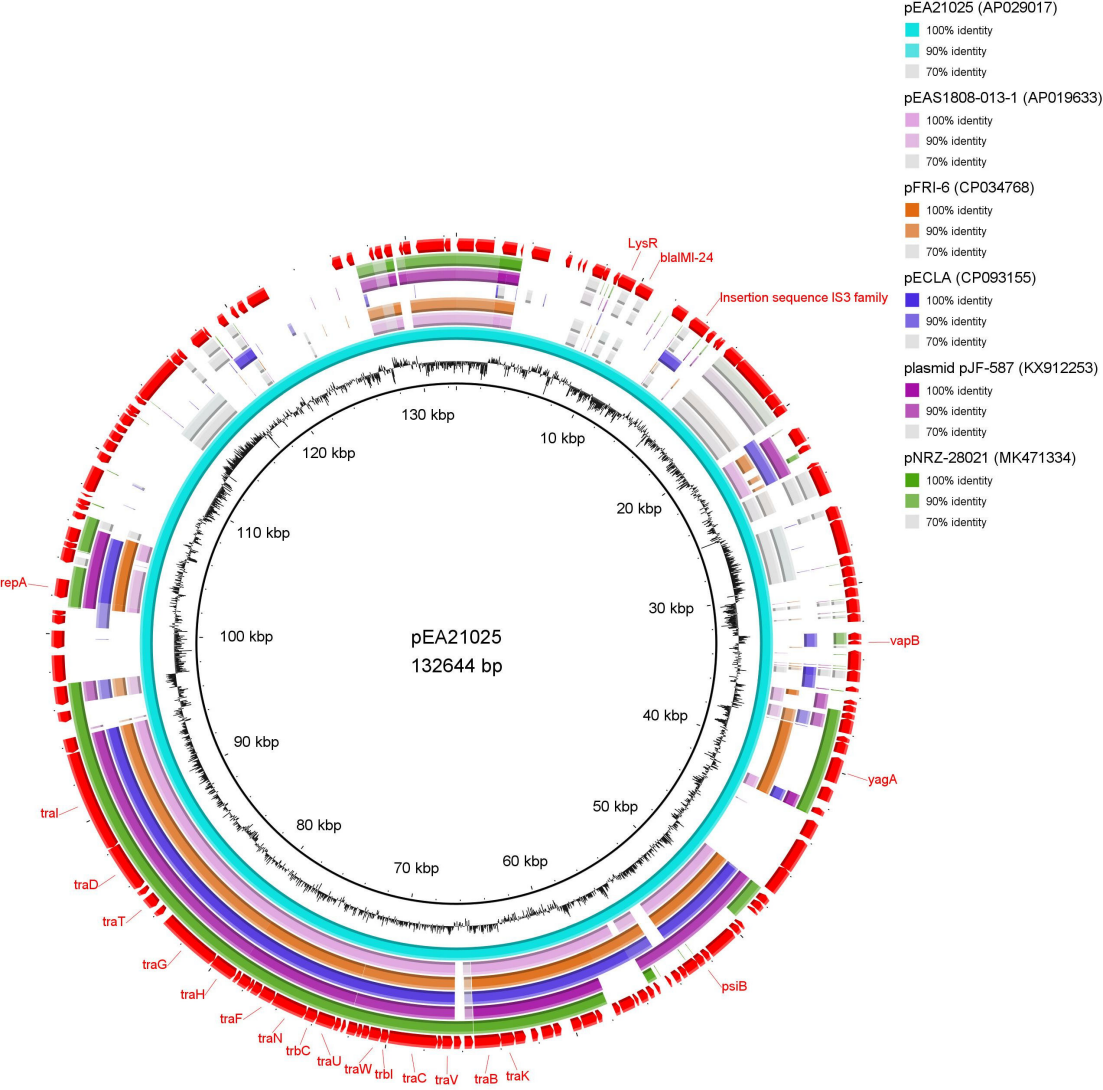
Circular comparison between the *bla*_IMI-24_-carrying plasmid pEA21025 and similar plasmids retrieved from the NCBI database. pEAS1808-013-1 (*Enterobacter asburiae*, IncFII type, accession no. AP019633, size 130,975 bp); pFRI-6 (*Enterobacter* sp., IncFII type, accession no. CP034768, size 125,786 bp); pECLA (*Enterobacter asburiae*, IncFII type, accession no. CP093155, size 159,417 bp); plasmid pJF-587 (*Enterobacter asburiae*, IncFII type, accession no. KX912253, size 108,672 bp); pNRZ-28021 (*Enterobacter cloacae*, IncFII type, accession no. MK471334, size 111,155 bp).

ResFinder v4.1 identified a plasmid-borne gene with 86.86% identity to *bla*_NMC-A_. BLASTN v2.12.0 showed 98.63% identity and 100% coverage for the gene to the *bla*_IMI-8_. This gene was thus registered as a new IMI-type variant, *bla*_IMI-24_ (accession: NG_079226). Furthermore, BLASTN v2.16.1 showed pEA21025 had the highest similarity (97.58%) to plasmid pEAS1808-013-1 from *E. asburiae* (47% query coverage, Fig. 1).

This study complies with the Infectious Disease Prevention Law.

## Data Availability

The genome and plasmid sequences of strain CRE21025 have been deposited at GenBank under accession numbers AP029016 and AP029017. The Illumina paired-end fastq and the Nanopore base-called fastq files are available in the Sequence Read Archive under accession numbers DRR437148, DRR511496, DRR505773, and DRR512896. The Illumina paired-end fastq files (DRR437148 and DRR511496) contain reads of varying lengths due to adapter trimming. The results of ResFinder, PlasmidFinder, BLASTN, and ANI analyses have been deposited in Figshare and are available at the following DOI: https://doi.org/10.6084/m9.figshare.28925723.
